# Role of Bacterial Exopolysaccharides as Agents in Counteracting Immune Disorders Induced by Herpes Virus

**DOI:** 10.3390/microorganisms3030464

**Published:** 2015-08-21

**Authors:** Concetta Gugliandolo, Antonio Spanò, Teresa L. Maugeri, Annarita Poli, Adriana Arena, Barbara Nicolaus

**Affiliations:** 1 Research Centre for Extreme Environments and Extremophiles, Department of Biological and Environmental Sciences, University of Messina, V.le F. Stagno d’Alcontres 31, 98166 Messina, Italy; E-Mails: cgugliandolo@unime.it (C.G.); aspan@unime.it (A.S.); tmaugeri@unime.it (T.L.M.); apoli@icb.cnr.it (A.P.); aarena@unime.it (A.A.); 2 Council of National Research (C.N.R.), Institute of Biomolecular Chemistry, Via Campi Flegrei 34, 80078 Pozzuoli, Italy; 3 Department of Human Pathology, Unit of Clinical Microbiology, Policlinico Universitario “G. Martino”, Via Consolare Valeria, 98125 Messina, Italy

**Keywords:** antivirals, Eolian Islands, exopolysaccharides, herpes simplex virus, immunomodulators, extremophiles, shallow marine vents

## Abstract

Extreme marine environments, such as the submarine shallow vents of the Eolian Islands (Italy), offer an almost unexplored source of microorganisms producing unexploited and promising biomolecules for pharmaceutical applications. Thermophilic and thermotolerant bacilli isolated from Eolian vents are able to produce exopolysaccharides (EPSs) with antiviral and immunomodulatory effects against Herpes simplex virus type 2 (HSV-2). HSV-2 is responsible for the most common and continuously increasing viral infections in humans. Due to the appearance of resistance to the available treatments, new biomolecules exhibiting different mechanisms of action could provide novel agents for treating viral infections. The EPSs hinder the HSV-2 replication in human peripheral blood mononuclear cells (PBMC) but not in WISH (Wistar Institute Susan Hayflic) cells line, indicating that cell-mediated immunity was involved in the antiviral activity. High levels of Th1-type cytokines were detected in PBMC treated with all EPSs, while Th2-type cytokines were not induced. These EPSs are water soluble exopolymers able to stimulate the immune response and thus contribute to the antiviral immune defense, acting as immunomodulators. As stimulants of Th1 cell-mediated immunity, they could lead to the development of novel drugs as alternative in the treatment of herpes virus infections, as well as in immunocompromised host.

## 1. Introduction

Marine hydrothermal systems (shallow and deep-sea vents) are considered “extreme environments” since they are characterized by high temperatures and unusual chemical conditions that are prohibitive for most organisms. Marine extreme habitats offer a rich source of undiscovered microorganisms able to produce unexploited metabolites for biotechnological purposes.

The Mediterranean Sea presents several shallow hydrothermal areas, of which those located at the Eolian Islands (Italy) and the Aegean Volcanic Arc (Greece) have been studied over more than a decade. These sites can be considered as excellent natural fields to investigate how microorganisms respond at elevated temperatures and salinity, high concentrations of CO_2_, and low pH, to isolate novel extremophiles, and to study the effects of the acidification and temperature increasing in the oceans. Due to the shallow depths, both light and hydrothermal energy support a complex microbial community living at these submarine hydrothermal vents, displaying a mixed origin of the primary productivity (phototrophic and chemotrophic). In particular, shallow hydrothermal systems of Eolian Islands (Italy), characterized by unusual conditions (high temperature, low pH, high concentrations of CO_2_, H_2_S, hydrocarbons, heavy metals, *etc*.) for the majority of organisms, represent easy accessible fields to isolate novel bacteria able to produce new biometabolites [1,2,3].

Thermophilic bacteria, defined by optimal growth temperature from 45 to 70 °C, isolated from Eolian Island shallow vents, offer attractive features for biotechnological purposes, as they are fast growing, and their biomolecules can be produced faster than those from mesophilic and psychrophilic counterparts [4]. Biomolecules from extremophilic bacteria are expected to possess unique characteristics (chemical composition and physicochemical properties) which are useful in responding to the increasing demand of new active bioproducts in medical and pharmaceutical applications [5]. Among these biomolecules, novel exopolysaccharides (EPSs) produced by *Bacillus licheniformis* strain B3-15 [6], *Geobacillus thermodenitrificans* strain B3-72 [7] and *B. licheniformis* strain T14 [8] have been reported among the few compounds until now derived from marine bacteria with antiviral and immunomodulatory activity [9,10,11,12,13,14].

This review focuses on the marine prokaryotes producers of EPSs exhibiting antiviral and immunomodulatory effects with particular attention paid to bacilli from Eolian shallow vents and their anti-herpes virus activities. Their immunostimulant and immunomodulatory effects make them potential novel drugs in the anti-herpes virus therapy.

## 2. Herpes Simplex Virus

The virus family of *Herpesviridae* comprises 130 different members of which nine have been described as human pathogen and on the basis of their biological characteristics have been divided in three subfamilies: *alpha-*, *beta-* and *gamma-herpesviridae* [15]. In the subfamily of *alpha-herpesviridae*, the common human pathogens herpes simplex viruses type-1 (HSV-1) and type-2 (HSV-2) cause a variety of mild or severe infections, mainly transmitted by close personal contact. HSV-1 is more frequently associated with oral-facial infections and encephalitis, whereas HSV-2 is responsible for gingivostomatitis, kerato-conjunctivitis, genital disease, encephalitis, and infections of newborns and immune-compromised patients. As reported by epidemiological surveys, the HSV infection rate has been continuously increasing around the world. After the primary infection, HSV tends to persist in the neuron of the ganglion, and the reactivation of latent HSV is very common during deficiency of immunity, causing recurrent herpes infection. Moreover, HSV-2 is a high risk factor for the acquisition of HIV infection and there is a synergistic relationship between HIV and HSV [16].

To counteract the development of viral diseases, the host immune response represents an important mechanism, based on the interaction among different immune cells, mediated by a complex network of cytokines and chemokines with pleiotropic effects [9,10,17]. Host immune response includes the release of cytokines such as interferon-gamma (IFN-γ), tumor necrosis factor-alpha (TNF-α) and other types of interferon, including IFN-α, with several antiviral effects. Interleukin 12 (IL-12), produced by monocytes and antigen-presenting cells, has been shown to stimulate IFN-γ -synthesis, therefore promoting the differentiation of naive CD4^+^ T cells to the Th1 phenotype and decreasing the synthesis of the type 2 cytokines IL-4 and IL-10 by CD4^+^ T cells [18]. IL-12 is known to induce an antiviral state in the cells (*i.e.*, *via* IFNs) or break virus-infected cells (*i.e*., *via* TNF-α), stimulating cytotoxicity and cytokine production (*i.e*., *via* IFN-γ) by T cells and natural killer (NK) cells, thus initiating the development of Th1 cells [19]. Moreover, IL-18 plays a critical role in the host defense against viral infection, promoting cell-mediated immunity via activation of NK and Th1-type cells [20]. Other than in inducing IFN-γ, IL-18 alone activates the cytotoxic T cells that are also known as CD8^+^ T cells, which play an important role in viral clearance, suggesting that this cytokine could influence the outcome of viral infection. The protective effect of IL-18 against HSV infection in a mouse model was reported [21]. HSV can indirectly counteract the phagocyte functions, namely by viral mechanisms of mimicry of cytokines and cytokine receptors [22,23]. As a consequence, networks of interactions between the host and viral molecules determine the nature and effectiveness of the host defense [24,25].

For the treatment of HSV infections, there are few drugs licensed, mainly based on topical antiviral therapies. Because these viruses establish a latent state in hosts, anti-herpes agents, such as nucleoside analogues, only control symptoms of disease or prevent outbreaks, and cannot cure the infections. The use of acyclovir and cogeners represents the treatment of choice in HSV infections, but their increasing use has led to the emergence of resistant viral strains. Drug resistance is an important clinical concern in the treatment of HSV infections, and the development of new antiviral agents exhibiting different mechanisms of actions, able to provide diverse antiviral actions in respect to the drugs commonly in use, is required, especially in prophylaxis treatments among transplanted patients and in immuno-compromised hosts. The development of new antiviral drugs that seek to combine immunotherapeutic intervention, as an adjunct to antiviral activity, is needed [26].

## 3. Marine Microbial Exopolysaccharides

EPSs are produced in response to biotic (like competition) and to abiotic (such as temperature, light intensity, pH, salinity) stress factors and/or as strategy of adaptation to extreme environments like in the case of acidophilic or thermophilic species of Bacteria and Archaea [27,28]. The physiological role of EPSs depends on the habitat and the natural conditions in which microorganisms live. Most of the functions ascribed to EPSs are of a protective nature. They could assist the microbial communities to endure extreme conditions of temperature, salinity and nutrient availability creating a boundary between the bacterial cells and their immediate environment [29]. In addition, they are involved in various cell functions, (*i.e.*, adhesion to abiotic and biotic surfaces, in the biofilm production), and as support in pathogenic and virulence mechanisms.

Marine organisms are known to be a promising source of therapeutic drugs. Most of the until now described EPSs are derived from Bacteria, whereas those from Archaea have been only recently reported [5,30,31]. Marine bacterial polysaccharides greatly differing in their composition and biological activities have been the subject of several reviews [27,32,33,34,35,36], however only a few of them focused on antiviral and immunomodulatory activities.

The antiviral activity against different viruses was reported for the polysaccharide isolated from the cyanobacterium *Arthrospira platensis* (formerly *Spirulina platensis*) [37]. This polysaccharide, containing spirulan-like molecules, showed a marked inhibition of human cytomegalovirus, herpes simplex virus type 1, human herpesvirus type 6 and human immunodeficiency virus type 1, but no inhibition was detected for Epstein-Barr virus and Avian influenza flu virus (influenza A virus) [37]. *Planococcus maitriensis* Anita I, isolated from the coastal area of Bhavnagar (India) [38], produced an EPS with oil-spreading potentiality comparable to that of Triton X100 and Tween 80, and therefore is suitable for the oil-recovery (bioremediation), as well as cosmetic applications. A marine *Enterobacter* *cloacae*, isolated from a sediment sample in India [39], was reported to produce an acidic EPS71a, with high content of uronic acids. This EPS showed emulsifying properties comparable to commercial gums. Recently, the homopolysaccharide (constituted by glucose) produced by *Pseudoalteromonas* sp. strain AM, isolated from a sponge collected in Red Sea has been described as a good emulsifier, comparable to natural gums [40]. This polymer showed antiviral properties against herpes simplex virus and fibrinolytic activity comparable to a pentosan sulphuric polyester, a fibrinolytic drug. Antiviral effects, evaluated in peripheral blood cells of a male with multiple myeloma (RPMI 8226 cells) infected with HSV-1, were reported for a sulphated polysaccharide produced by the marine *Pseudomonas* strain WAK-1 [41].

EPSs derived from extremophiles have been reported over the last decades, and their greatly variable composition, structure, biosynthesis and functional properties have been extensively studied, even if only a few of them have been industrially developed. Components most commonly found in marine EPSs are monosaccharides, such as pentoses as d-Arabinose, d-Ribose and d-Xylose, hexoses as d-Glucose, d-Galactose, d-Mannose, d-Allose, l-Rhamnose and l-Fucose, amino sugars such as d-Glucosamine and d-Galactosamine or uronic acids such as d-Glucuronic acids and d-Galacturonic acids. Organic or inorganic substituents, for example sulphate, phosphate, acetic acid, succinic acid and pyruvic acid, may also be present [5].

In the Archaea domain, *Thermococcus* *litoralis* produces an EPS constituted only by mannose, representing a peculiar feature among prokaryotes since the synthesis of mannan-like polymers is typically of eukaryotes [42]. Moreover, this EPS, containing sulphate (1%–2%) and phosphorus (1.5%–4.5%), showed *in vitro* activity against human immunodeficiency virus type I [43].

A novel EPS TA-1 isolated from *Thermus aquaticus* YT-1 displayed immunomodulatory effects in murine macrophage and in human monocyte cell lines [44].

The most common EPSs producers are halophilic prokaryotes belonging to the genus *Halomonas*, such as *H. maura*, *H. anticariensis*, *H. eurihalina* and *H. ventosae*. These strains, isolated from hypersaline environments, are able to produce EPSs rich in sulphate and uronic acids, responsible for their good gelifying properties [45,46,47,48,49]. In particular, the EPS produced by *H. maura* (mauran), other than possessing interesting physicochemical and rheological properties (*i.e.*, high viscosity, heavy metals binding, thixotropic and pseudoplastic features) showed immunomodulator effects and antiproliferative activities on human cancer cells, which are encouraging in pharmaceutical applications [50].

Although other marine bacteria have been reported to be producers of EPSs with biotechnological potentiality, the EPSs produced by the three bacilli isolated from vents of the Eolian Islands are among the few biopolymers described to exhibit antiviral and immunomodulatory effects.

## 4. Eolian Shallow Vents and Bacterial EPS Producers

**Figure 1:**
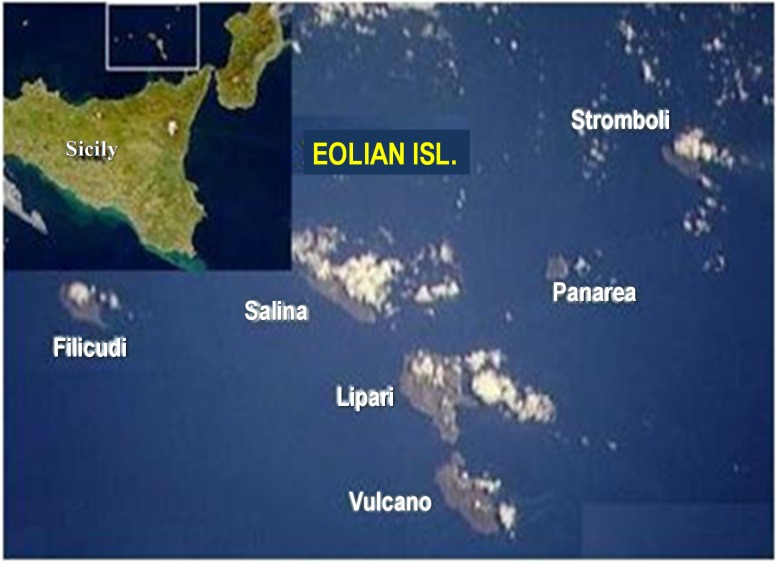
Eolian Archipelago (Italy).

The Eolian Archipelago (Tyrrhenian Sea, Italy), located 25 km north of Sicily, consisting of seven main islands (Figure 1), hosts numerous shallow hydrothermal vents related to both active and extinct volcanism, at a depth allowing investigations by scuba divers.

The main characteristics of the vent sites and the isolated strains producing EPSs are reported in Table 1. Strains were isolated from thermal fluid samples collected from shallow, submarine vents off Eolian Islands, Italy. In detail, *Bacillus licheniformis* strain B3-15 [6] and *Geobacillus thermodenitrificans* strain B3-72 [7] were isolated from a marine spring of Vulcano Island at Porto di Levante, whereas *Bacillus licheniformis* strain T14 was isolated from Bottaro, Panarea Island [8].

A comparison of the differential phenotypic characters of *B. licheniformis* strain B3-15, *B. licheniformis* strain T14 and *G. thermodenitrificans* strain B3-72 are shown in Table 2.

*B. licheniformis* strain B3-15 and strainT14 were more halophilic, than G. *thermodenitrificans* strain B3-72, and in particular strain T14 showed the highest optimum of NaCl concentration of growth (5%). Differently, *G. thermodenitrificans* strain B3-72 was the most thermophilic with an optimum of growth of 65 °C. All strains grew aerobically in a wide range of temperature, pH and NaCl%, indicating a great physiological versatility that allow them to adapt to the severe environmental conditions.

**Table 1:** Physical and chemical characteristics of thermal fluids emitted from the shallow hydrothermal vents off Eolian Islands (Italy) and related isolates.

Site	Depth (m)	T (°C)	pH	Conductivity (mS/cm)	Strain
Bottaro, Panarea Island	8.0	55	5.4	42.90	T14
Porto di Levante, Vulcano Island	0.7	70	5.2	-	B3-15, B3-72

**Table 2:** Differential phenotypic characteristics of the bacilli producing exopolysaccharides, isolated from Eolian shallow vents.

Phenotypic Characteristics	*Bacillus licheniformis*		*Geobacillus thermodenitrificans*
	Strain B3-15	Strain T14	Strain B3-72
Growth temperature (°C)	25–60	25–60	45–70
Optimum temperature (°C)	45	50	65
Growth pH	5.5–9	4–10	6–9
Optimum pH	7	8	7
Growth NaCl	0–7	2–10	0–2
Optimum NaCl	2	5	0
Reduction of nitrate to nitrite	−	+	+
Catalase	+	+	−
Oxidase	+	+	−
**Hydrolysis of:**			
Starch	−	+	−
Tween 20	+	−	+
Tween 80	+	−	−
**Acid production from:**			
l-arabinose	−	+	−
d-galactose	−	+	−
d-glucose	+	+	−
Inositol	−	+	−
d-mannitol	+	+	−
d-sorbitol	−	+	−
Methyl-α-d-glucopyranoside	−	+	−
Amygdalin	−	+	−
Arbutin	−	+	+
Salicin	−	+	+
d-cellobiose	+	+	+
d-maltose	−	+	+
d-melibiose		−	+
d-saccharose	−	+	+
d-melezitose	+	−	−
d-raffinose	−	−	+
Potassium 2-ketogluconate	+	−	+
**Acid production from:**			
Potassium 5-ketogluconate	+	−	+
**Antibiotic resistence to:**			
Bacitracin	+	−	−
Polymyxin B	+	+	−

+: positive; –: negative.

## 5. EPSs Production and Characterization

**Figure 2:**
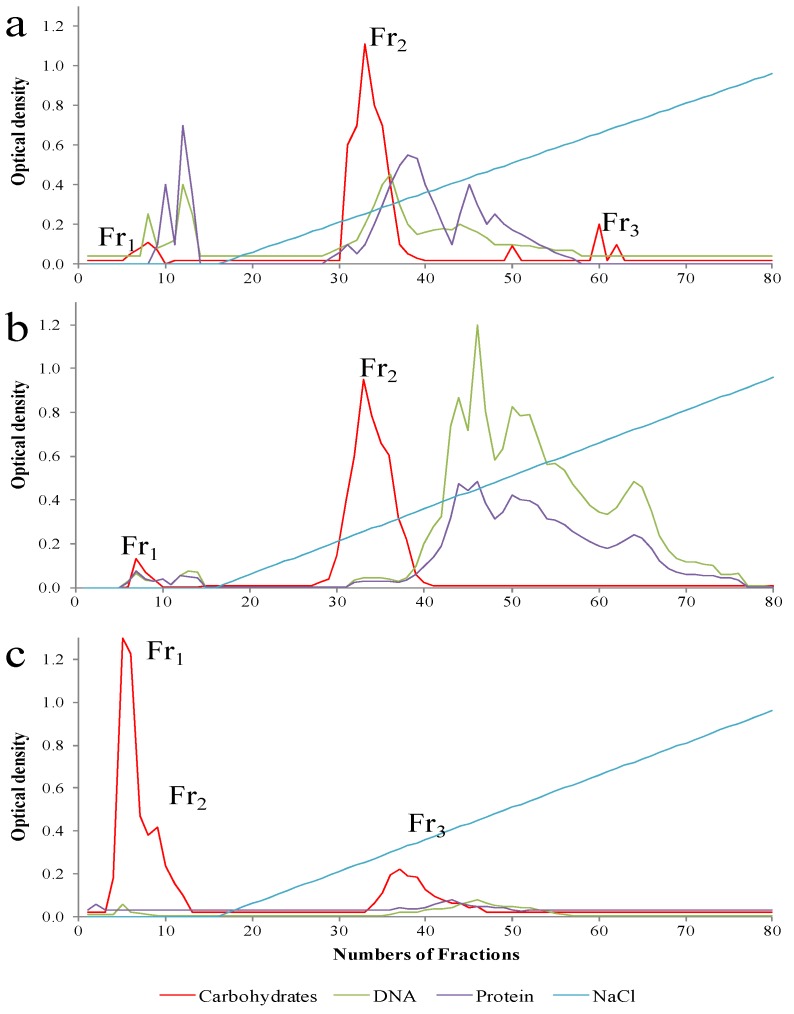
Elution profiles from DEAE-Sepharose CL-6B of EPSs fractions produced by *Bacillus licheniformis* strain B3-15 (**a**); *Geobacillus thermodenitrificans* strain B3-72 (**b**); *Bacillus licheniformis* strain T14 (**c**).

The amounts of crude biopolymer obtained was 165 mg·L^−1^ for strain B3-15, 70 mg·L^−1^ for strain B3-72 and 366 mg·L^−1^ for strain T14, obtained after optimization of the EPS-production by testing different concentrations of sugars using as sole carbon and energy sources [6,7,8]. After purification, two or three fractions were obtained (Figure 2) of which the more abundant EPS-B3-15 Fr2 (EPS2-B3-15), EPS-B3-72 Fr2 (EPS2-B3-72) and EPS-T14 Fr1 (EPS1-T14) were further investigated.

Carbohydrate content was the highest (99%) for the EPS1 T14, whereas 66% and 80% were obtained from EPS2-B3-15 and EPS2-B3-72, respectively (Table 3).

**Table 3:** Chemical characterization of EPSs produced by bacilli isolated from Eolian shallow vents.

Properties	EPS2-B3-15	EPS2-B3-72	EPS1-T14
EPS production (mg·L^−1^)	165	70	366
Sugar-media	Glucose	Glucose	Sucrose
Carbohydrate content (%)	66	80	99
Protein content (%)	5	3	1.2
Molecular weight (KDa)	600	400	1000
Monosaccharide composition (ratio of relative portion)	Man	Man/Glu (1:0.2)	Fru/Fuc/Glu/GalN/Man (1.0:0.75:0.28:trace:trace)
Saccaride repeating unit	Tetrasaccharide	Trisaccharide	Trisaccharide
Anomeric configuration	Manno-pyranosidic	Manno-pyranosidic	Manno-pyranosidic

Monosaccharide analysis of EPSs revealed that EPS2-B3-15 was mainly composed by mannose, EPS2-B3-72 by mannose and glucose in a relative molar ratio of 1:0.2. Hydrolyzed EPS1-T14 was composed by fructose/fucose/glucose/galactosamine/mannose in a relative molar ratio of 1.0:0.75:0.28:trace:trace (Table 3).

Hydrolysis of the polysaccharide was performed with 0.5 N or 2 N trifluoroacetic acid (TFA) at 120 °C for 2 h. The sugar components were identified by both Thin Layer Chromatography (TLC) and high pressure anion exchange-pulsed amperometric detector (HPAE-PAD)-Dionex equipped with a CarboPac PA1 column ((DIONEX, Thermo Scientific, Illkirch, France), and the sugars were eluted isocratically with 16 mM NaOH using standards for identification and calibration curves.

**Figure 3:**
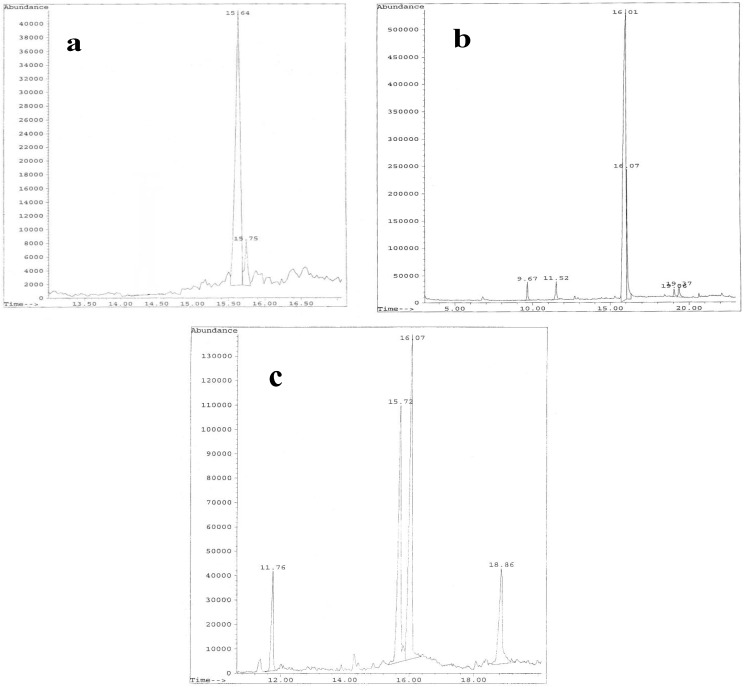
GC-MS of hydrolysed EPSs fractions produced by *Bacillus licheniformis* strain B3-15 (**a**); *Geobacillus thermodenitrificans* strain B3-72 (**b**); *Bacillus licheniformis* strain T14 (**c**).

**Figure 4:**
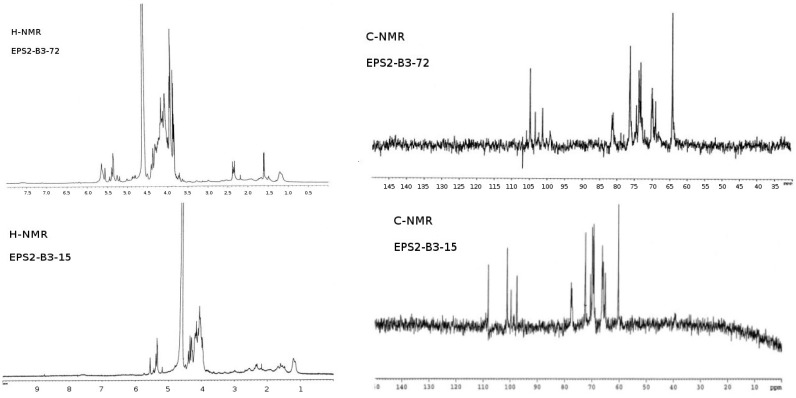

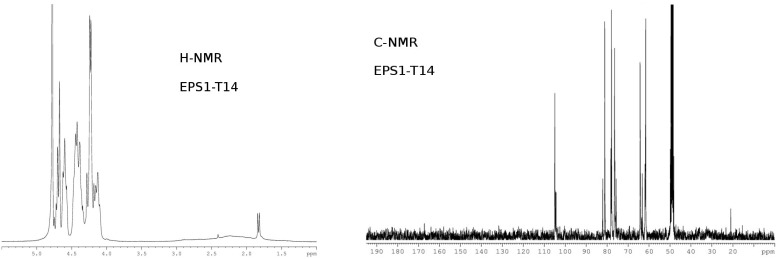
^1^H-NMR and ^13^C-NMR spectra of EPSs fractions.

For glycosyl analysis by Gas Chromatography-Mass Spectroscopy (GC-MS), using a Hewlett Packard 5890A gas chromatograph fitted with a FID detector (San Diego, CA, USA), the sample was mildly hydrolyzed with 1% AcOH at 100 °C for 4 h. The products were reduced with NaBD_4_ and acetylated using Ac_2_O and pyridine at 100 °C for 30 min. The presence of glucitol and mannitol were detected by using GC-MS, as previously described [31]. After hydrolysis of each fraction, GC-MS of EPSs produced by the three strains are shown in Figure 3. A comparison of ^1^H-NMR and ^13^C-NMR (nuclear magnetic resonance) from the purified EPSs are reported in Figure 4. The ^1^H-NMR from EPS2-B3-15 spectrum showed four anomeric signals at δ 5.19 (s), δ 5.35 (s), δ 5.38 (s) and δ 5.56 (s) all of them have a little coupling constant (about 1 Hz) probably due to a *manno* configuration and one signal at δ 5.44 (s) with coupling constant (about 3.7 Hz) indicating the occurrence of a *gluco-galacto* configuration. These data suggested a tetrasaccharide repeating unit. ^13^C-NMR spectrum confirmed this observation: the anomeric carbon region contains four signals at δ 97.2 (sharp), δ 99.1, δ 100.1 and δ 108 of equal intensity and a low intensity signal at δ 98.5. The remaining signals (^1^H-NMR and ^13^C-NMR) were attributable to ring protons and carbons and confirm the presence of a pyranosidic hexose. From the comparison of the chemical shift values in ^1^H and ^13^C-NMR spectra and the little values of coupling constant of H1-H2 (less than 1 Hz), we could conclude that the EPS2 of strain B3-15 was a polymer with a tetrasaccharide repeating unit, essentially constituted by sugars having a *manno pyranosidic* configuration.

The ^1^H-NMR of EPS2-B3-72 (Figure 4) showed three major anomeric signals at δ 5.00 (s), δ 5.05 (s) and δ 5.22 (s), all of them have a small coupling constant (about 1 Hz) probably due to a *manno* configuration. This observation seemed to be confirmed by ^13^C-NMR spectrum (Figure 4): the anomeric carbon region contained three signals at δ 101.2, δ 103.4 and δ 104.8. Remaining signals (^1^H-NMR and ^13^C-NMR) were attributable to ring protons and carbons and confirmed the presence of a pyranosidic hexose. After comparing the chemical shift values in ^1^H and ^13^C spectra and the small values of the coupling constant of H1-H2 (less than 1 Hz), we concluded that the EPS2-B3-72 was a trisaccharide repeating unit, essentially constituted by sugars having a *manno pyranosidic* configuration.

The low-field region of ^1^H-NMR spectrum of EPS1-T14 (Figure 4) exhibited different signals of which only three showed connectivity to anomeric carbon atoms. In particular, the anomeric proton signals at δ 4.70, 4.67, and 4.62 ppm were identified by their correlation with carbon signals at δ 104.41, 104.54 and 104.97 ppm, respectively, displayed in ^13^C spectrum (Figure 4). In addition, the presence of the signal at δ 61.65 ppm in ^13^C spectrum confirmed the β-backbone structure of the polymer.

The most common inorganic substituents are sulphate and phosphate groups. The absence of these substituting groups were assessed by means of Fourier-transform infrared spectroscopy (FTIR) and nuclear magnetic resonance (NMR) in Eolian EPSs.

**Figure 5:**
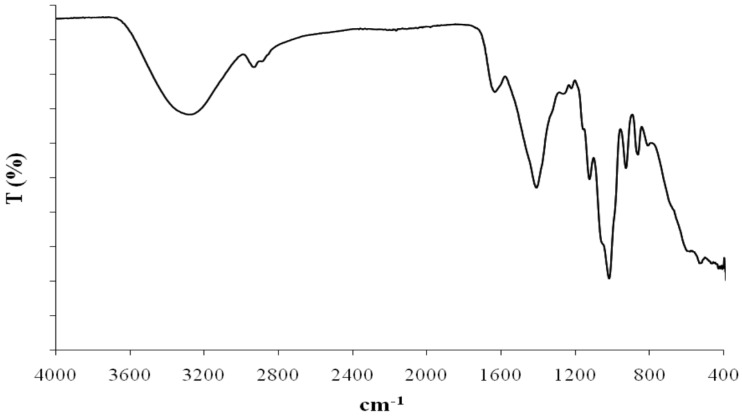
IR spectrum of the EPS1 produced by *B. licheniformis* strain T14.

Fourier transform-infrared spectroscopy (FT-IR) is another spectral technique that was applied to EPS1-T14 structural investigation (Figure 5). IR spectrum showed a strong signal at 3400 cm^−1^ assignable to OH stretching, a signal at 2900 cm^−1^ attributable to CH stretching and signals at 1490 and 1045 cm^−1^ attributable to CH and OH deformation, respectively. The band at 1635 cm^−1^ could be assigned to a *N*-acetyl group or to the stretching vibration of C=O groups. Signal at 1159 cm^−1^ could be attributed to the stretching vibrations of C–O–C.

The elucidation of the EPSs structures are very important to clarify the physico-chemical and biological properties of these biopolymers, and to attribute and in some case to predict the biotechnological applications of the EPS-producer microorganisms or of their products [6,7,8]. Several chemical characteristics of polysaccharides (*i.e.*, charge and nature of carbohydrates components) may influence their biological activity. As revealed by chemical analyses, the novel EPSs are composed by neutral monosaccharides. The EPS1 from T14 showed the highest molecular weight, being 1000 KDa, the other EPSs were in a quite similar order of magnitude. EPS2-B3-15 and EPS2-B3-72 were mainly composed by mannose, and the smallest repetitive unit was found in EPS2 from B3-72, possessing a trisaccharide unit, whereas the repetitive unit in B3-15 was a tetrasaccharide.

Differently from the others EPSs, EPS1 from the haloalkaliphilic, thermophilic *B. licheniformis* strain T14 was a heteropolymer displaying fructose and fucose as major monosaccharides, which confer to the biopolymer interesting chemical and rheological characteristics [8]. Polysaccharides containing fucose have been reported to have biological properties, such as anticarcinogenic and anti-inflammatory agents, useful in the treatment of rheumatoid arthritis, in age-related pathologies accompanied by tissue loss, in acceleration of wound healing and as hydrating and anti-ageing additives [33,34]. Moreover, polysaccharides rich in fucose can be used as a source of fucose monosaccharides which are otherwise difficult to achieve by chemical synthesis or through the expensive and laborious chemical extraction from plant tissues.

## 6. EPSs Anti-Herpes Virus Activity

The cytotoxicity of the different EPSs towards peripheral blood mononuclear cells (PBMC) is reported in Table 4. The results showed a dose-dependent cytotoxicity in PBMC, comparable to those obtained on WISH cells (data not showed). All EPSs were noncytotoxic at the concentration of 300 μg·mL^−1^, whereas EPS1-T14 was also noncytotoxic at the concentration of 400 μg·mL^−1^, and therefore EPSs at these concentrations were selected for subsequent assays.

**Table 4:** Cytotoxicity percentage on PBMC cells 48-h post-EPSs treatments. Values represent the means of three experiments ±S.D.

EPS	Cytotoxicity Percentage on PBMC Cells					
	200 μg·mL^−1^ *	300 μg·mL^−1^ *	400 μg·mL^−1^ *	500 μg·mL^−1^ *	600 μg·mL^−1^ *	700 μg·mL^−1^ *
EPS2-B3-15	0	0	12 ± 2.0	29 ± 5.0	48 ± 2.9	57 ± 4.5
EPS2-B3-72	0	0	4 ± 0.6	12 ± 2.2	29 ± 6.8	40 ± 7.3
EPS1-T14	0	0	0	12 ± 1.1	31 ± 4.9	60 ± 1.9

* EPS concentration.

All EPSs were able to hinder HSV-2 replication at the utilized concentrations. In particular, the ability to inhibit HVS-2 replication in PBMC, expressed as logarithm, was higher for EPS2-B3-15 (0.82) compared with EPS2-B3-72 (0.49) and EPS1-T14 (0.63). Moreover, EPSs treatment on WISH cells did not show any significant inhibition of HSV-2 replication (data not shown).

## 7. Immunomodulator and Immunostimulant Effects

The antiviral effect observed on PBMC but not on WISH cells prompted us to investigate if the antiviral activity of EPSs could be related to the immune response involved in the controlling viral replication. Therefore, the production of Th1 cytokines (IFN-γ, IFN-α, TNF-α, IL-12 and IL-18) was evaluated on PBMC treated with EPSs, infected or not with HSV-2 (Table 5).

Results showed high levels of cytokines detected in supernatants of EPSs treated PBMC. It is noteworthy that, at the concentration of 300 µg·mL^−1^, EPS1-B3-15 induced the highest amount of IFN-γ, IFN- α, TNF-α, IL-12 (*P* < 0.05), whereas EPS2-B3-72 was the highest inducer of IL-18 (*P* < 0.05). On the other hand, when PBMC was treated with EPSs and then infected with HSV-2, cytokine production was down-regulated in all the experimental conditions (*P* < 0.05) (Table 5), with a marked reduction of Th1 cytokine production.

In order to understand if this down-regulation could be due to the presence of Th2-type cytokines in PBMC supernatants, the release of IL-4 and IL-10 was analyzed. In Table 6 are reported the results of IL-4 and IL-10 production after treatment with EPSs by PBMC infected or not with HSV-2.

EPSs treatment did not trigger uninfected-PBMC to release IL-4 and IL-10, which are strong hallmarks of Th2 response. Whereas, the HSV-2 infection of untreated PBMC induced an appreciable amount of IL-4 and IL-10. Furthermore, the HSV-2 infection of PBMC treated with EPS1-T14 induced lower amount of IL-4 compared with that induced by HSV-2 (23 ± 5.8 *vs.* 41 ± 6.1) (*P* < 0.05). On the contrary, EPS1-T14 treatment was not able to significantly counteract the IL-10 cytokine production.

**Table 5:** Production of Th-1 cytokines (pg·mL^−1^) at 48 h post-EPSs treatments by PBMC and under the effect of HSV-2 infection (in grey). Values are expressed as the mean of four experiments ±S.D.

Inducer	IFN-γ	IFN-α	TNF-α	IL-12	IL-18
None	˂0.06	˂3.1	˂0.13	˂2.1	˂9.2
HSV-2	˂0.08	˂3.6	˂0.12	˂2.2	˂8.9
EPS2-B3-15 (300 μg·mL^−1^)	165 ± 19 *^,†^	480 ± 76 *^,†^	2151 ± 328 *^,†^	420 ± 78 *^,†^	140 ± 35 *^,†^
EPS2-B3-72 (300 μg·mL^−1^)	115 ± 18 *^,†^	116 ± 13 *^,†^	1980 ± 101 *^,†^	320 ± 49 *^,†^	183 ± 29 *^,†^
EPS1-T14 (300 μg·mL^−1^)	58 ± 13 *^,†^	45 ± 3 *^,†^	610 ± 43 *^,†^	128 ± 19 *^,†^	49 ± 3 *^,†^
EPS1-T14 (400 μg·mL^−1^)	105 ± 28 *^,†^	108 ± 25 *^,†^	1310 ± 73 *^,†^	358 ± 69 *^,†^	86 ± 3 *^,†^
EPS2-B3-15 (300 μg·mL^−1^) + HSV-2	79 ± 24	295 ± 93	780 ± 98	115 ± 28	84 ± 22
EPS2-B3-72 (300 μg·mL^−1^) + HSV-2	61 ± 9	42 ± 5	680 ± 71	122 ± 17	95 ± 13
EPS1-T14 (300 μg·mL^−1^) + HSV-2	27 ± 2	29 ± 2	301 ± 28	57 ± 11	23 ± 8
EPS1-T14 (400 μg·mL^−1^) + HSV-2	37 ± 2	26 ± 2	317 ± 88	166 ± 20	28 ± 10

* Significantly different (*P* ˂ 0.05) compared with untreated control; ^†^ Significantly different (*P* ˂ 0.05) compared with exopolymer treated PBMC and HSV-2 infected.

**Table 6:** Production of Th2 cytokines (IL-4 and IL-10) (pg·mL^−1^) at 48 h post-EPSs treatments by PBMC and under the effect of HSV-2 infection (in grey). Values are expressed as mean of four experiments ±S.D.

Inducer	IL-4	IL-10
None	˂0.1	˂0.5
HSV-2	41 ± 6	35 ± 5
EPS2-B3-15 (300 μg·mL^−1^)	˂0.1	˂0.5
EPS2-B3-72 (300 μg·mL^−1^)	˂0.1	˂0.5
EPS1-T14 (400 μg·mL^−1^)	˂0.1	˂0.5
EPS2-B3-15 (300 μg·mL^−1^) + HSV-2	˂0.1 *	˂0.5 *
EPS2-B3-72 (300 μg·mL^−1^) + HSV-2	˂0.1 *	˂0.5 *
EPS1-T14 (400 μg·mL^−1^) + HSV-2	23 ± 6	37 ± 2

* Significantly different (*P* ˂ 0.05) compared with PBMC infected with HSV-2.

## 8. Conclusions

Drug resistance is an important clinical concern in the treatment of HSV infection, and the development of new antiviral agents, able to provide diverse antiviral actions in respect to the drug commonly in use, is required. Therefore, the search for less expensive, readily available and less toxic alternative agents to control and prevent HSV infection and its transmission is an urgent matter.

Many sulphated polysaccharides have been proven to possess very strong antiviral activity [51,52]. Sulphated EPSs, such as mannan sulphate, chondroitin sulphate, heparin, and sulphoevernan, may be responsible for eukaryotic cell protection from viruses. The antiviral effects of sulphated EPSs are due to their structural features and not only to their charge density and chain length. In fact, as demonstrated *in vivo*, these EPSs are able to inhibit the attachment of the virion to the surface of the host cell [53]. On the other hand, due to their well-known anticoagulant activity, sulphated polysaccharides may present undesirable side effects [53]. It is noteworthy that EPSs from Eolian bacilli with antiviral and immunomodulatory activity are not sulphated.

It is now generally agreed that a complex network of cytokines with pleiotropic effects orchestrate the immune response of the host against several viral infections [9,10,12]. On the other hand, it is well established that viruses have devised multiple mechanisms in manipulating cytokine production, to survive as successful pathogens. Furthermore, HSV-2 is able to down-regulate the production of Th1 cytokines in PBMC, which represent the first line of defense against viral infections, the so-called pro-inflammatory host response, and at the same time to stimulate Th2 cytokines response in order to evade the immune system of the host [9,10,12,17].

EPSs treatment induced high amounts of Th1 cytokines (IFN-γ, IFN-α, TNF-α, IL-12 and IL-18) by PBMC, leading to a restriction of viral replication via the induction of antiviral state in neighboring cells (*i.e.*, IFNs) or the destruction of virus-infected cells (*i.e.*, TNF-α and IL-18). Interestingly, EPS2-B3-15 treatment induced the highest levels of Th1 cytokines, and at the same time showed the best antiviral activity compared with the other EPSs tested. Hence, these results confirm our hypothesis that the EPSs could be able to restore the immune response involved in controlling viral replication [54,55,56]. As stimulators of Th1 cell-mediated immunity, these biopolymers could be used as therapy in immunocompromised hosts. Therefore, it could be speculated that the treatment with the studied EPSs represent an innovative therapeutic approach during HSV-2 infection, able to improve immune surveillance of PBMC toward viral infection, by tipping the balance in favor of Th1 immune response. As immunomodulatory agents, EPSs can indirectly act on HSV-2 replication, through the up-regulation of cell-mediated immunity, by inducing a Th1 cytokine pattern, as usually involved in the antiviral response. These findings suggest that the water-soluble, non-cytotoxic, not-sulphated EPSs derived from Eolian strains could play an important role in determining the effectiveness of host immunity against viral infections.

## 9. Future Perspectives

For their growth characteristics (wide range of temperature, pH and salinity), Eolian bacilli may be considered to be producers of valuable compounds for pharmaceutical applications. In future researches, the effects of their EPSs on the complex network of cytokines, orchestrating the immune response of the host during a viral infection, will be analyzed through novel assays, such us Real-Time Polymerase Chain Reaction (RT-PCR), able to directly detect and quantify the expression of the genes involved in the regulation of cytokine production.
